# Disease Modifying Effects of the Spider Toxin Parawixin2 in the Experimental Epilepsy Model

**DOI:** 10.3390/toxins9090262

**Published:** 2017-08-25

**Authors:** Lívea Dornela Godoy, José Luiz Liberato, Marcus Vinícius Batista Celani, Leonardo Gobbo-Neto, Norberto Peporine Lopes, Wagner Ferreira dos Santos

**Affiliations:** 1 Laboratório de Neurobiologia e Peçonhas (LNP), Faculdade de Filosofia, Ciências e Letras de Ribeirão Preto, Universidade de São Paulo, Av. Bandeirantes, 3900, CEP 14040-901 Ribeirão Preto, São Paulo, Brazil; liveagodoy@usp.br (L.D.G.); jll@usp.br (J.L.L.); sucram.swe@hotmail.com (M.V.B.C.); 2 Instituto de Neurociências e Comportamento (INEC), Av. do Café, 2450, CEP 14050-220 Ribeirão Preto, São Paulo, Brazil; 3 Núcleo de Pesquisas em Produtos Naturais e Sintéticos (NPPNS), Faculdade de Ciências Farmacêuticas de Ribeirão Preto, Universidade de São Paulo, Av. do Cafe s/n, CEP 14040-903 Ribeirão Preto, São Paulo, Brazil; gobboneto@yahoo.com.br (L.G.-N.); npelopes@fcfrp.usp.br (N.P.L.)

**Keywords:** lithium-pilocarpine model, temporal lobe epilepsy, Parvalbumin, GABA transporter inhibitor, neuroprotection, hippocampus, Spider toxin, Parawixia bistriata, Tiagabine

## Abstract

(1) **Background:** Temporal lobe epilepsy (TLE) is the most common type of epilepsy in adults. It is also the one with the highest percentage of drug-resistance to the current available anti-epileptic drugs (AED). Additionaly, most antiepileptic drugs are only able to control seizures in epileptogenesis, but do not decrease the hippocampal neurodegenerative process. TLE patients have a reduced population of interneuronal cells, which express Parvalbumin (PV) proteins. This reduction is directly linked to seizure frequency and severity in the chronic period of epilepsy. There is therefore a need to seek new therapies with a disease-modifying profile, and with efficient antiepileptic and neuroprotective properties. Parawixin2, a compound isolated from the venom of the spider *Parawixia bistriata*, has been shown to inhibit GABA transporters (GAT) and to have acute anticonvulsant effects in rats. (2) **Methods:** In this work, we studied the effects of Parawixin2 and Tiagabine (an FDA- approved GAT inhibitor), and compared these effects in a TLE model. Rats were subjected to lithium-pilocarpine TLE model and the main features were evaluated over a chronic period including: (a) spontaneous recurrent seizures (SRS), (b) neuronal loss, and (c) PV cell density in different regions of the hippocampus (CA1, CA3, DG and Hilus). (3) **Results:** Parawixin2 treatment reduced SRS frequency whereas Tiagabine did not. We also found a significant reduction in neuronal loss in CA3 and in the *hilus regions* of the hippocampus, in animals treated with Parawixin2. Noteworthy, Parawixin2 significantly reversed PV cell loss observed particularly in DG layers. (4) **Conclusions:** Parawixin2 exerts a promising neuroprotective and anti-epileptic effect and has potential as a novel agent in drug design.

## 1. Introduction

It is estimated that one third of the population living with epilepsy has Temporal Lobe Epilepsy (TLE), accounting for nearly 700,000 patients in the United States. TLE is also the most frequent type of epilepsy among adult patients with focal epilepsy and it is the one with the highest refractoriness index (patients who do not respond to current antiepileptic drugs [1,2]). Hippocampal sclerosis—also known as mesial temporal sclerosis—is the most common histopathological feature associated with TLE. This neurodegenerative process followed by gliosis represents a clinical feature associated with uncontrolled drug-resistant epileptic seizures [3].

After the injury, aberrant synaptic contacts appear, excitatory neurotransmission is enhanced, and abnormal mossy fiber sprouting induces hyperexcitability [4]. Also, recurrent seizures themselves may lead to the formation of more epileptic circuits. Complementary to the enhancement of excitatory neurotransmission, these circuits operate with a reduced dendritic inhibition that may facilitate the spread of excitatory inputs to main body cells [5,6].

In TLE patients, there is a decrease in the number and intensity of an important interneuronal cell population which expresses the Parvalbumin (PV) protein [7]. PV is a calcium-binding protein that is responsible for buffering calcium concentrations and for promoting limbic synchronization. In the chronic period, PV interneuron loss is directly linked to seizure frequency and severity [8,9]. Unlike glutamatergic synapses—which present considerable plasticity—there is little evidence that lost GABAergic interneurons can be replaced [5]. Since strong evidence supports that PV decreases in response to epileptogenic activity in the hippocampus, PV cells may represent a potential cell subpopulation that could be targeted for anti-epileptic therapy [8].

Although GABAergic interneurons are severely compromised in TLE, seizures are not only generated in response to the reduction of GABAergic neurotransmission [10]. Moreover, a profound change during the epileptogenic process should be considered, in which GABA functional properties are impaired [11]. Considering that seizure manifestation involves a synchronized neuronal hyperexcitability, it is therefore plausible that epilepsy results from an imbalance between excitatory and inhibitory neurotransmission [12].

Drugs that enhance GABA neurotransmission can control seizures in epileptic patients [10], usually either by stimulating release, activating receptors, or by inhibiting transport and enzymes that degrade GABA [1,13,14,15,16]. Hence, seeking novel compounds that target GABA neurotransmission could be helpful, since currently available compounds are either not effective or they present serious side effects such as sedation, tolerance and hepatotoxicity [17]. In this sense, drugs that inhibit GABA reuptake are very promising once it results in enhanced GABA availability in the synaptic cleft that is already synthesized.

The development of novel disease-modifying strategies is a topic of high interest in the Epilepsy field [18]. Neurotoxins isolated from arthropode venoms are potential sources for drug discovery and are interesting tools that have been shown to affect neuronal mechanisms with high affinity and selectivity. Many pharmaceutical companies have developed venom-based drug discovery programs or use venom-derived molecules for target validation [19].

Our group has isolated a molecule from the venom of the spider *Parawixia bistriata*, known as Parawixin2 (formerly FrPbAII) [20]. This compound has a potent GABA uptake inhibitor effect on cerebrocortical and retina synaptosomes [20]. Parawixin2 was demonstraded to be neuroprotective in an experimental glaucoma model [20], and intracerebroventricular (i.c.v) administration of this compound inhibits seizures induced by different chemical convulsants in acute models in rats, including pilocarpine [21,22,23,24]. However, this compound was never examined in a chronic model of epilepsy. The need for new therapies for epilepsy and the promise of compounds that target the GABA transport makes Parawixin2 a potential compound to be further studied in the context of epilepsy. This study aimed to investigate the efficiency of Parawixin2 in altering epileptogenesis in a chronic model of TLE.

## 2. Results

### 2.1. High Perfomance Liquid Cromatography and Dereplication

**Figure 1:**
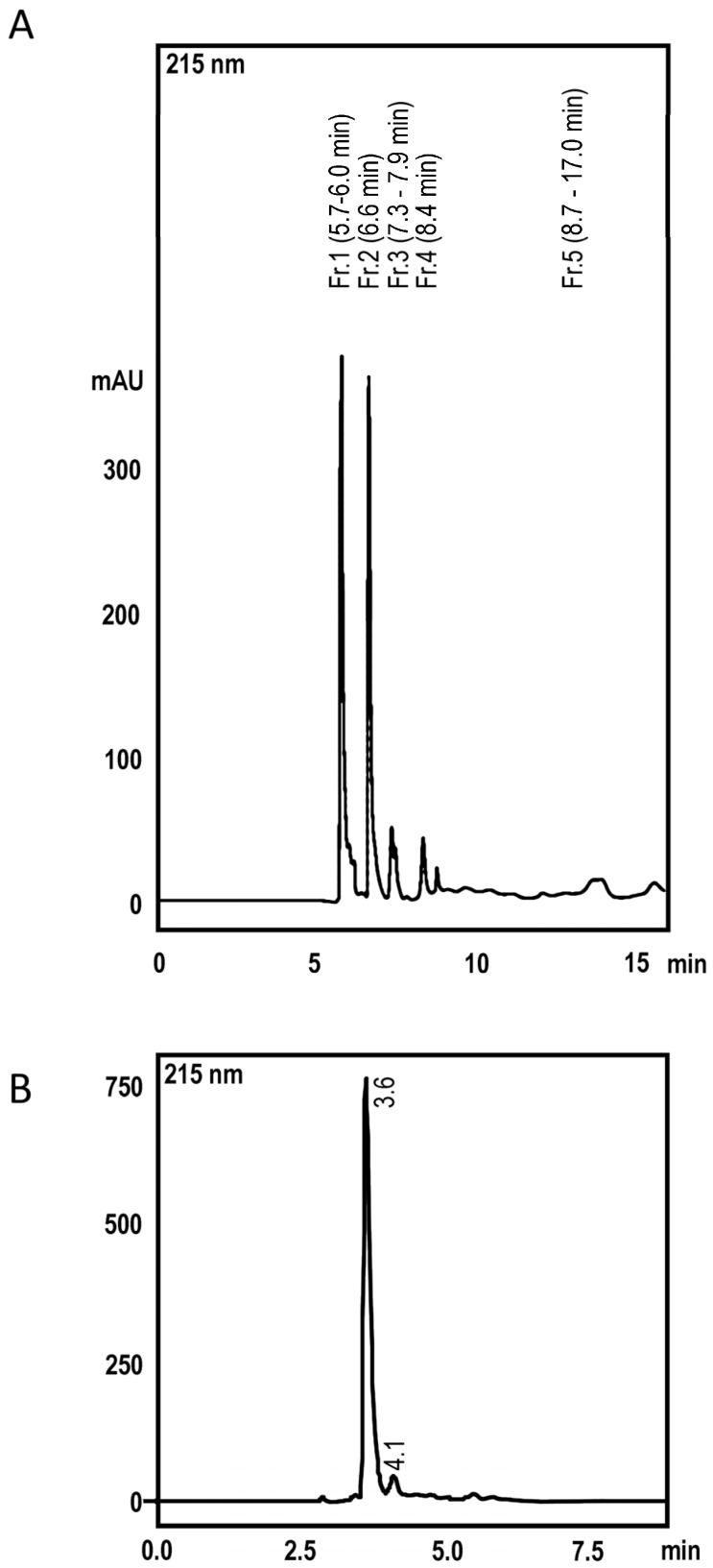
Parawixin2 was obtained by two chromatographic isolation (**A**). Chromatogram obtained for Fr.2 (r.t. 6.6min in the first fractionation) at 215 nm. (**B**). Fr2 fraction was submitted to a second fractionation and Parawixin2 was obtained at r.t. 4.1 min.

**Figure 2:**
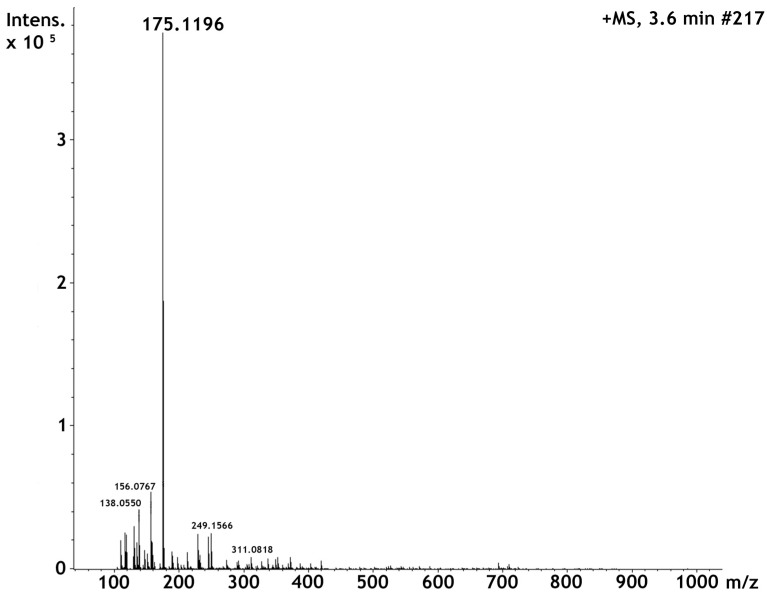
High Resolution ESI-MS spectrum of Parawixin2 showing the protonated molecule [M + H] ^+^ ion peak at *m*/*z* 175.1196. The calculated molecular formula for [M + H] ^+^ is C_6_H_15_N_4_O_2_^+^ (0.6 ppm error).

To confirm the identity of Parawixin2 we used LC-MS to compare retention time and UV, MS and MS/MS spectra with those previously reported (Beleboni et al., 2006). Parawixin2 was isolated by two sequenced chromatography purifications. The first chromatogram profile obtained for the elution of Parawixin 2 is shown in Figure 1. The fractionation at 215 nm allowed the identification of Pb2 at 6.6 min retention time (r.t.). The second chromatogram is identical to the report by Beleboni et al., 2006 and Parawixin2 was obtained at the retention time (r.t.) 4.1 min. UV max for the isolated compound was 200 nm. High Resolution ESI-MS showed the protonated molecule [M + H] ^+^ at *m*/*z* 175.1196, giving us the calculated molecular formula C_6_H_15_N_4_O_2_^+^ for [M + H] ^+^ with a 0.6 ppm error in ESI-MS (Figure 2). MS/MS fragmentation was essentially the same reported by Beleboni et al. (2006). Beside of the loss of ammonia (*m*/*z* 158), the loss of water (*m*/*z* 157) was observed too. In addition, the loss of a guanidine group (*m*/*z* 116) and the protonated guanidine itself (*m*/*z* 60) was detected.

### 2.2. Anticonvulsant Effects of Parawixin2 on Spontaneous Recurrent Seizures (SRS)

TLE models based on pilocarpine administration present the advantage of experimentally reproducing three distinct phases of the epileptogenic process. The first phase corresponds to an initial injury that can be represented by Status Epilepticus (SE). This phase is followed by a latent period, when the epileptogenesis process happens. The third phase is characterized by Spontaneous Recurrent Seizures (SRS), and epileptogenesis process still occurs [25].

In this part of study, we analyzed the pharmacological intervention on the third phase, with the evaluation of the following experimental groups: SE + Vehicle, SE + TGB (SE + Tiagabine) and SE + Pwx2 (SE + Parawixin2).

Administration of lithium-pilocarpine resulted in limbic seizures that reached SE in 67% (*n* = 20) of animals, whereas the remaining 33% (*n* = 10) were not classified as SE, and therefore were excluded from the study. Latency to SE was approximately 30 min. Among those animals that reached SE only one died.

Latency to Spontaneous Recurrent Seizures (SRS) was 15 ± 1.9 days (mean ± SEM) (*n* = 17). Most SRS occurred when animals were asleep, and the most frequent behaviors were oral-facial and head myoclonic movements with forelimb myoclonic/extensions. Animals that did not present SRS were excluded from the study (*n* = 2).

**Figure 3:**
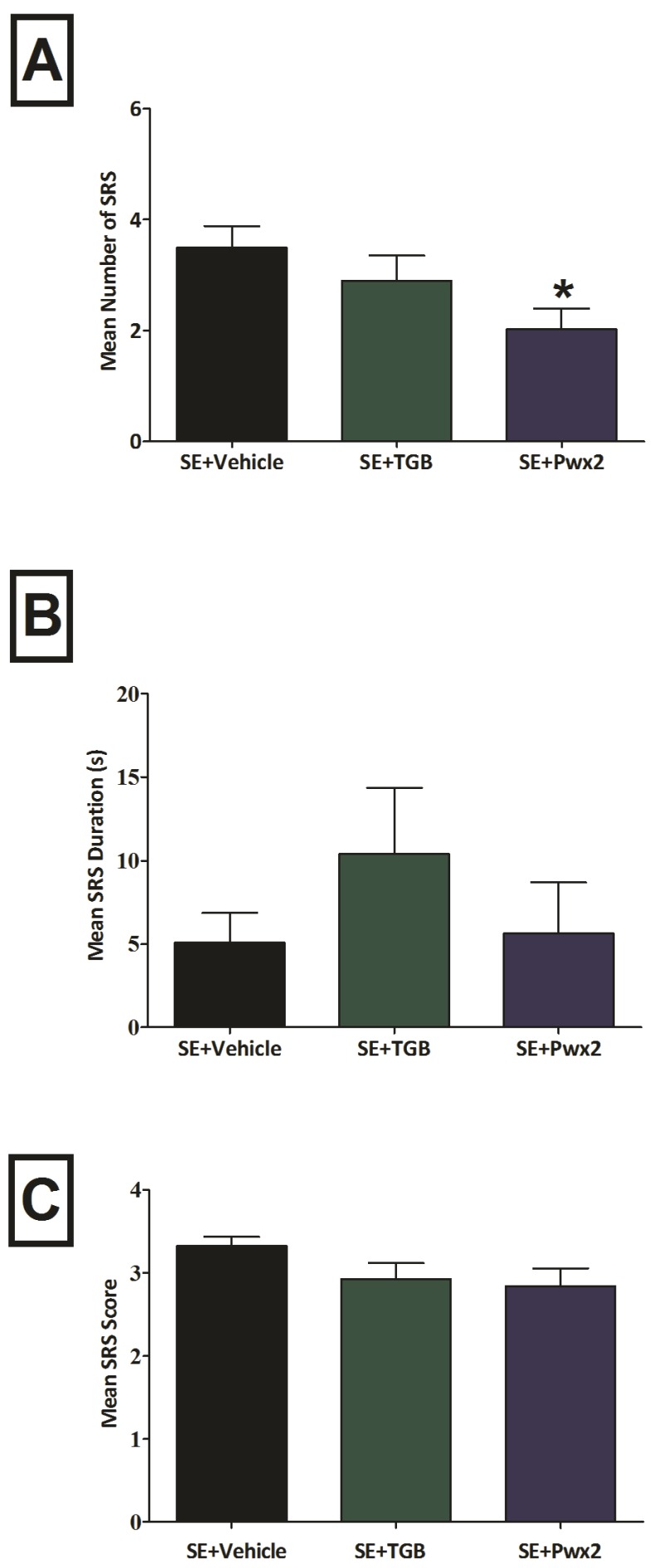
Parawixin2 treatment reduced total seizure frequency. (**A**). Analysis of spontaneous recurrent seizure in (**A**). Mean frequency. (**B**). Mean Duration and (**C**). Mean Score in experimental groups Status Epilepticus (SE) + Vehicle, SE + TGB (SE + Tiagabine) and SE + Pwx2 (SE + Parawixin2) * *p* < 0.05 compared to SE + Vehicle.

We observed a statistical difference for the mean number of seizures [F_(2,14)_ = 3.570; *p* < 0.05] (Figure 3A). Post hoc analyses revealed that Parawixin2 treatment significantly reduced the number of seizures, in comparison to the vehicle treated group (*p* < 0.05). Significant differences were not observed for seizure duration [F_(2,14)_ = 0.0944; *p* = 0.9104] (Figure 3B) nor for seizure severity [F_(2,14)_ = 0.2718; *p* = 0.7662] (Figure 3C).

**Figure 4:**
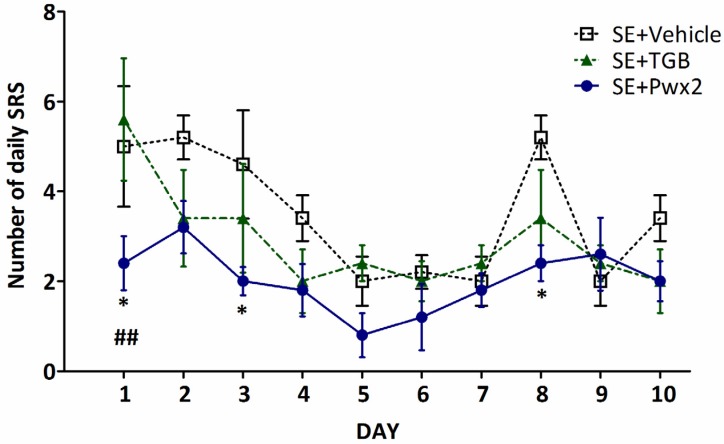
Effects of Parawixin2 and Tiagabine treatment on daily analysis of seizure frequency. Analysis of spontaneous recurrent seizure frequency during the 10 days of treatment in SE + Vehicle, SE + TGB (SE + Tiagabine) and SE + Pwx2 (SE + Parawixin2) groups * *p* < 0.05 compared to SE + Vehicle, ^##^ *p* < 0.01 compared to SE + TGB.

The frequency of daily seizures was compared among the three experimental groups. Two-way ANOVA revealed a statistical difference for treatment factor [F_(2,12)_ = 3.349; *p* < 0.0069] (Figure 4). The group treated with Parawixin2 presented a lower number of daily seizures, with post hoc analyses showing a significant difference on the 1st, 3rd, and 8th days compared with the Vehicle group (*p* < 0.05). Also, Parawixin2 treatment is significantly different from Tiagabine treatment on the 1st day (*p* < 0.01).

Time was also significantly different in the two way ANOVA [F_(2,108)_ = 6.568; *p* < 0.0001]. There was a significant difference in post hoc analyses for time when comparing Vehicle and Tiagabine treated animals. Detailed description of the multiple comparison analyses are presented in Supplementary Table S1. Vehicle group was significantly different over time. Tiagabine administration also resulted in a significant difference over the 10-day period, but to a much less extent than vehicle group.

Parawixin2 treatment exhibited a significant effect at reducing and stabilizing seizure frequency over the period of 10 days and did not present significant difference in any of the multiple comparisons (Figure 4 and Suplementary Table S1). Significant differences for interaction [F_(18,108)_ = 1.354; *p* = 0.1702] were not observed.

**Figure 5:**
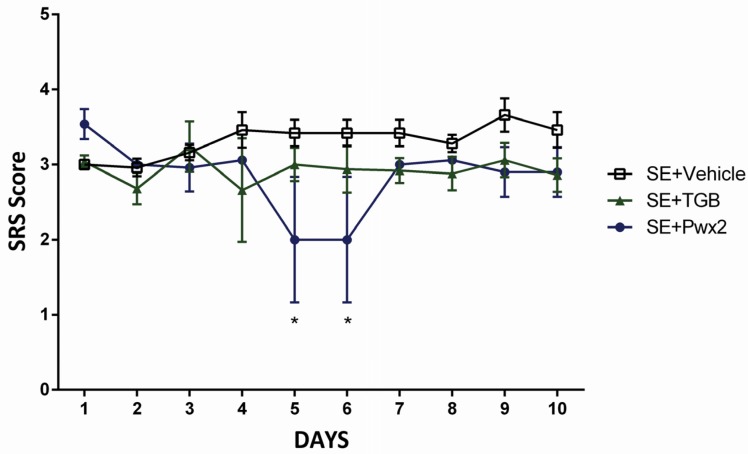
Effect of Parawixin2 treatment on seizure score. Analysis of spontaneous recurrent seizure score (Racine scale) during the 10-day treatment period in SE + TGB (SE + Tiagabine) and SE + Pwx2 (SE + Parawixin2) * *p* < 0.05 compared to SE + Vehicle.

The analyzis of daily SRS score through two way ANOVA revealed that, although there is no statistical significance for treatment [F_(2,12)_ = 6.372; *p* = 0.1623], after performing the post hoc analysis it was shown that Parawixin2 presented a significant lower number of seizures in comparison to vehicle group, on the 5th and 6th days (*p* < 0.05) (Figure 5).

There was no statistical difference either for time factor [F_(9,108)_ = 0.503; *p* = 0.6830] or interaction [F_(18,108)_ = 1.545; *p* = 0.0886].

Regarding SRS duration, there was no difference for treatment [F_(2,12)_ = 0.8991*;* *p* < 0.4327] time [F_(18,108)_ = 1.091; *p* = 0.3753] or interaction [F_(18,108)_ = 0.6542; *p* = 0.8480] (data not shown).

### 2.3. Histopathological Analysis

#### 2.3.1. Hippocampal Neuronal Protection with Parawixin2 Treatment

We analyzed the most important subregions of hippocampus linked to cell death in epileptogenesis (including the first and third region of *Cornus of Amon* (CA), named CA1 and CA3 respectively, and the granular and hilar region of the Dentate Gyrus (DG).

**Figure 6:**
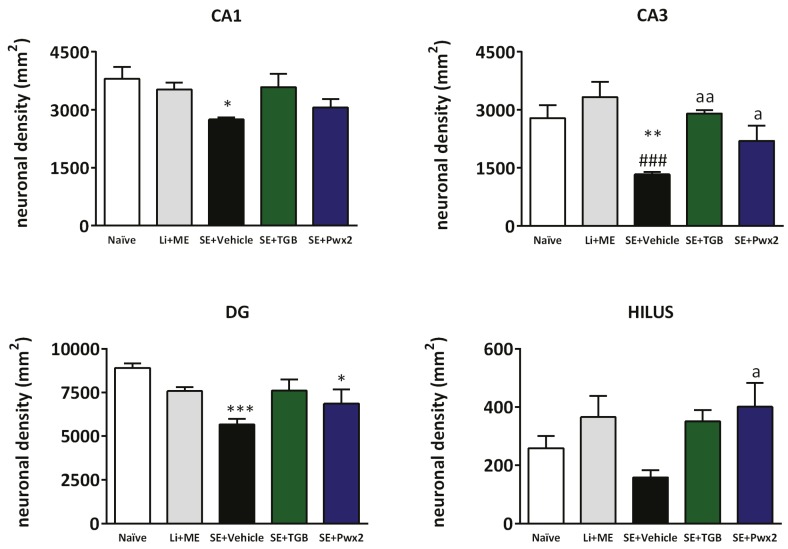
Histogram of neuronal density in four hippocampus regions of Naïve, Li + Me (Lithium + Metylscopolamine), SE + Vehicle, SE + TGB (SE + Tiagabine) and SE + Pwx2 (SE + Parawixin2) groups. SE induced neuronal loss in CA1, CA3, DG and hilus. Parawixin2 was neuroprotective in CA3 and hilus, while Tiagabine protected only in CA3 layer * *p* < 0.05, ** *p* < 0.01 and *** *p* < 0.001 compared to Naïve group; ^###^ *p* < 0.001 compared to Li + Me group; ^a^ *p *< 0.05 and ^aa^ *p* < 0.01 compared to SE + Vehicle group.

Quantification analyses of neuronal density by one-way ANOVA revelead a significant difference in cell density in all hippocampal regions: CA1 [F_(4,24)_ = 3.497; *p* = 0.0255], CA3 [F_(4,24)_ = 6.968; *p* = 0.001], DG [F_(4,24)_ = 6.019; *p* = 0.002] and hilus [F_(4,24)_ = 2.980; *p* = 0.0428] (Figure 6).

The vehicle treated group showed a significant reduction in neuronal density when compared with healthy control groups. In the CA3 region, we observed layer disorganization and vacuolization induced by SE. Layer disorganization in the hilus is followed by an extensive neuronal loss. As shown in Figure 6, we found significant reduction in CA1 (*p* < 0.05) and the granular layer of the DG (*p* < 0.001), as compared to Naïve. In the CA3 layer, vehicle group had significantly lower number of neurons when compared to Naïve (*p* < 0.01) and Li + Me (*p* < 0.001).

Conversely, animals treated with Parawixin2 had a significantly higher number of viable neurons in the CA3 and *hilus* regions (both *p* < 0.05) in comparison with the vehicle group. Also, treatment with tiagabine resulted in a significantly higher number of viable neurons in CA3, when compared with vehicle treated animals (*p* < 0.01).

**Figure 7:**
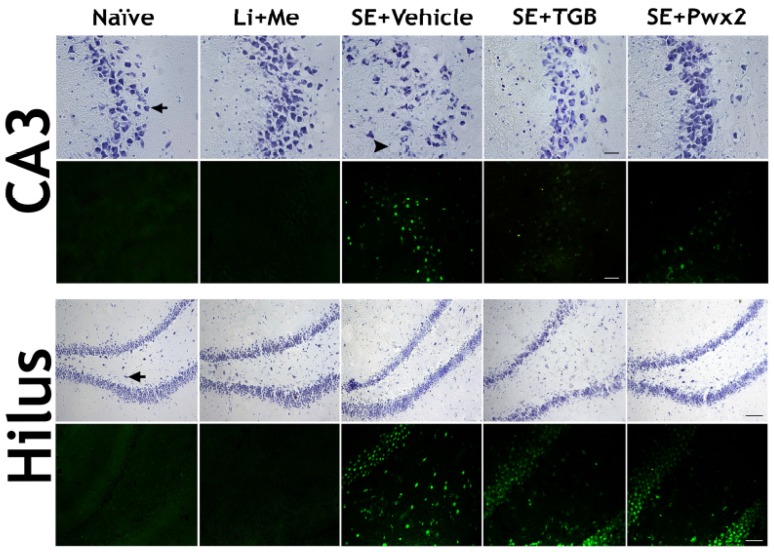
Parawixin2 presented neuroprotective effect on CA3 and hilus regions. Representative images of CA3 and hilus transversal sections of CA3 and hilus stained for Nissl (upper rows) and Fluoro-Jade C staining (lower rows). Experimental groups are Naïve, Li + Me (Lithium + Metylscopolamine), SE + Vehicle, SE + TGB (SE + Tiagabine) and SE + Pwx2 (SE + Parawixin2), respectively. In CA3 there is layer disorganization and vacuolization induced by SE. Layer disorganization in hilus is followed by an extensive neuronal loss. In both regions, an intense Fluoro-Jade C staining is present. Treatment with Tiagabine and Parawixin2 for 10 days prevented CA3 disorganization and Parawixin2 prevented neurodegeneration both in CA3 and hilus. Black arrow indicates for viable neurons; Head arrow indicates for pyknotic with vacuolization. Scale bar: represents 20 μm. In both regions, an intense Fluoro-Jade C staining is present. Treatment with Tiagabine and Parawixin2 for 10 days prevented CA3 disorganization. Also, Parawixin2 treatment reduced FJC+ cells in both CA3 and hilus regions (Figure 8).

Representative images of hippocampal sections are shown in Figure 7. All hippocampal layers in the vehicle-treated group presented neuronal degeneration resulting from SE, as layer disorganization and thickening, along with the presence of pyknotic nuclei and vacuolization.

Regarding neurodegeneration, healthy control groups (Naïve and Li + Me) presented no Fluorojade-C positive (FJC+) cells in hippocampal layers, whilst the SE vehicle-treated group showed a large number of FJC+ neurons (Figure 7).

A qualitative analysis of the histological data also revealed that Parawixin2 treatment preserved layer organization and thickness, similar to control groups. Furthermore, this treatment resulted in a reduction in the number of pyknotic nuclei and cell vacuolization.

**Figure 8:**
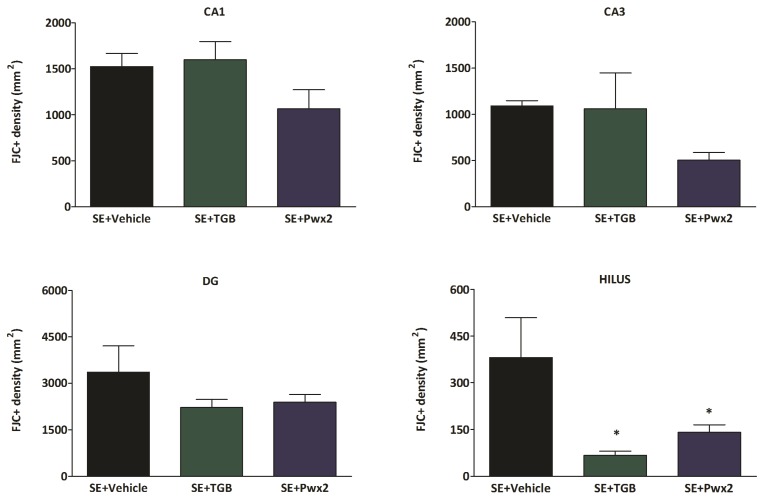
Histogram of Fluoro-Jade C positive neurons in four hippocampal regions analyzed in the following groups: SE + Vehicle, SE + TGB (SE + Tiagabine) and SE + Pwx2 (SE + Tiagabine). SE + Vehicle showed intense Fluoro-Jade C staining in CA1, CA3, DG and hilus. Tiagabine and Parawixin2 treatments were selectively neuroprotective in hilus. * *p* < 0.05 compared to SE + Vehicle.

Complementary to these results, we also demonstrated that rats subjected to SE and treated with Parawixin2 had fewer FJC+ neurons. Statistical analyses of FJC labeling revealed that there is significant difference only in the hilar region [F_(2,19)_ = 5.286; *p* = 0.0164], and that in both groups treated with Tiagabine and Parawixin2 there was a significant reduction in comparison with the vehicle group (*p* < 0.05). In the other regions, no statistical differences were observed in the CA1 region [F_(2,19)_ = 2.434; *p* = 0.1176], CA3 [F_(2,19)_ = 2.162; *p* = 0.1496] or DG [F_(2,19)_ = 1.445; *p* = 0.2634] (Figure 8).

#### 2.3.2. Parawixin2 Treatment Preserved Parvalbuminergic (PV) Cells

**Figure 9:**
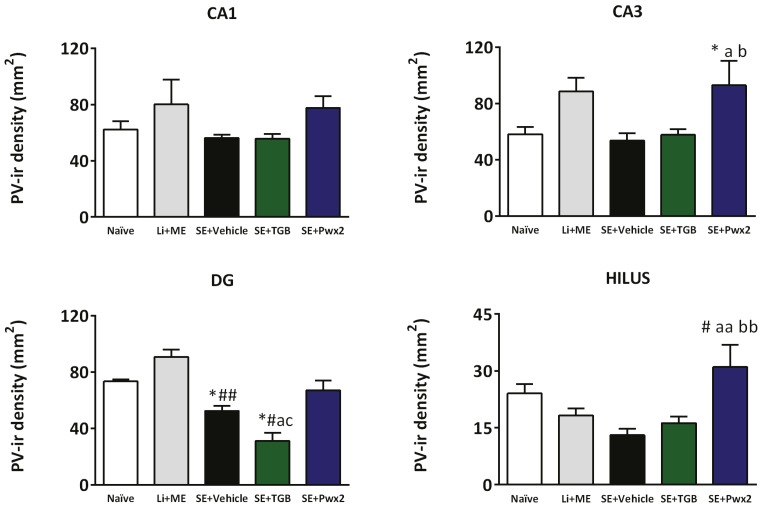
Histogram of parvalbumin positive cell density in four hippocampus regions, in Naïve, Li + Me (Lithium + Metylscopolamine), SE + Vehicle, SE + TGB (SE + Tiagabine) and SE + Pwx2 (SE + Parawixin2) groups. In the Dentate Gyrus (DG) there is evidence of parvalbumin positive cell reduction in epileptic animals, which is decreased with Tiagabine treatment. In both the CA3 and hilus regions, Parawixin2 treatment prevented Parvalbuminergic cell loss and, moreover, it resulted in significantly higher density of parvalbuminergic neurons, in comparison to control groups. * *p* < 0.05 compared to Naïve; ^#^ *p* < 0.05 and ^##^ *p* < 0.01 compared to Li + ME; ^a^ *p* < 0.05 and ^aa^ *p* < 0.01 compared to SE + Vehicle; b *p* < 0.05 and bb *p* < 0.01 compared to SE + TGB; c *p* < 0.05 compared to SE + Pwx2.

Histopathological analysis of the hippocampus included the evaluation of parvalbumin (PV) staining. One-way ANOVA of immunohistochemistry quantification for parvalbumin showed a significant difference in cell density in CA1 [F_(4,27)_ = 2.921; *p* = 0.00433], CA3 [F_(4,27)_ = 4.398; *p* = 0.0087], the granular layer of the DG [F_(4,27)_ = 16.52; *p* < 0.0001] and the hilus [F_(4,27)_ = 5.189; *p* = 0.0040] (Figure 9). There was a significant loss of Parvalbumin immunoreactive (PV-ir) cells only in the granular layer of the DG (*p* < 0.05 and *p* < 0.01 compared to Naïve and Li + Me respectively). Additionaly, this loss was intensified by Tiagabine treatment (*p* < 0.05 compared with all other groups).

In contrast, treatment with Parawixin2 significantly increased the number of PV-ir cells in CA3 (*p* < 0.05) and the hilus (*p* < 0.01), both compared with Vehicle- and Tiagabine- treated groups. Parawixin2 treatment also resulted in a significantly higher number of PV-ir cells, when compared with the naïve group in CA3 (*p* < 0.05) and Li + Me group in hilus (*p* < 0.01).

**Figure 10:**
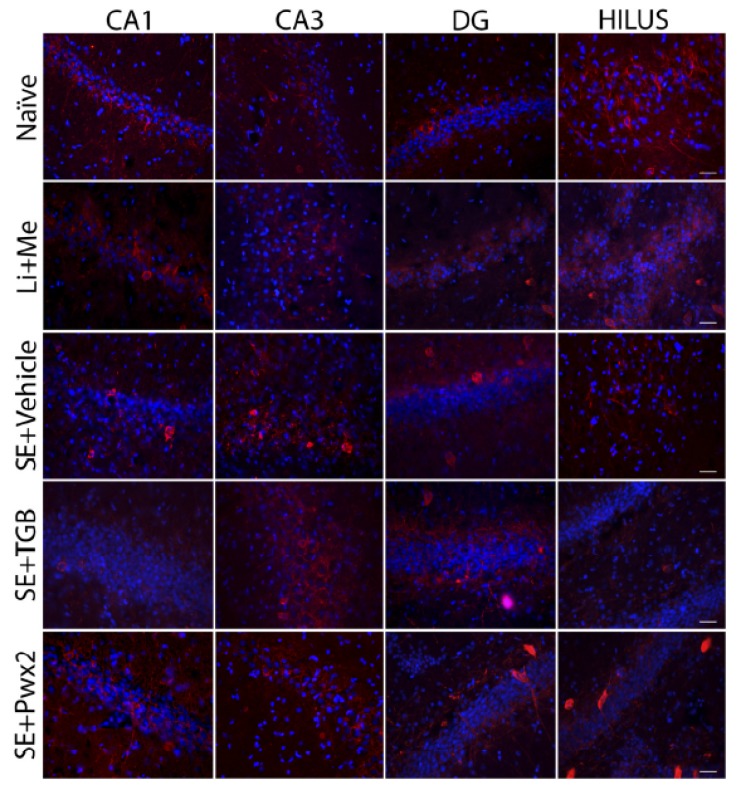
Representative images of transverse sections from hippocampal regions, with Parvalbumin in red and DAPI in blue staining. Columns represent groups Naïve, Li + Me (Lithium + Metylscopolamine), SE + Vehicle, SE + TGB (SE + Tiagabine) and SE + Pwx2 (SE + Parawixin2), respectively; and rows represent CA1, CA3, DG and the hilus, respectively. Parvalbumin positive cells appear morphologically different in many hippocampal areas in SE + vehicle group. Groups treated with Tiagabine and Parawixin2 showed higher density of positive parvalbumin cells and better-preserved morphology. Scale bar represents 20 μm.

Representative images of hippocampal sections are shown in detail in Figure 10. It can be seen that PV-ir interneurons in hippocampal layers of Naïve and Li+Me groups presented oval shapes and intact appearance, mainly located in the vicinity of hippocampal layers evaluated, including surrounding neurons. Some extensions of PV-ir neurons radiate near the interneuron body in control groups, but also present long projections, with varied and distant connections with other layers.

In contrast, in vehicle-treated animals, PV-ir appeared to be altered in hippocampal layers, with morphological features, such as appearing more rounded, which may be present in deformed shape and located more distantly from hippocampal cells. PV-ir projections, when apparent, were restricted to layers where cell bodies were located. These interneurons may lie near regions with low neuronal density, most evidently in the CA1 and DG regions. In those regions, it is worth noting that there was a marked decrease in the number of these interneurons, or when present, they were marginalized and closer to the granular layer.

The groups treated with Tiagabine and Parawixin2 presented a higher number of PV-ir cells and a well-preserved morphology. We highlight that, in the Parawixin2 group, the DG and hilus layers presented high PV-ir neurons densities with defined shapes and a strong staining, in contrast with the vehicle-treated group, in which those neurons permeated through the layers also presenting long projections.

## 3. Discussion

### 3.1. Effects of Parawixin2 and Tiagabine Treatment on SRS

The pilocarpine model used in this work is one of the most studied animal models of epilepsy, and it mimicks many features of the human epileptic condition, such as SRS and mesial temporal sclerosis [26,27]. Also, this study analyzed the SRS in the chronic phase, which is considered to be a consistent model by which to study pharmacoresistance [8,28]. Our study reproduced the behavioural and histopathological data reported in other lithium-pilocarpine studies. Also, we observed that seizure frequency oscillated over time in vehicle treated group. Previous studies have described such variability on seizure frequency and severity in the chronic phase [8,27,29].

In this study, Parawixin2 treatment promoted an antiepileptic effect, by reducing the frequency and severity of seizures. The anticonvulsive effect of Parawixin2 had been previoulsy shown in acute epilepsy experimental models [21,22,23,24], including the pilocarpine model [18]. Previous studies also established that Parawixin2 crosses the blood-brain barrier [20], therefore, this compound has therapeutic potential. Additionaly, it was shown to have an anxiolytic-like effect in the elevated plus maze and light/dark box apparatus model [22]. Moreover, rats administered with this compound had no cognitive impairment in the Morris water maze, no changes in general, and locomotor activity in the open field [23], demonstrating lack of toxicity in these models.

However, in this model, the treatment with Tiagabine did not result in antiepileptic properties in the chronic period (60–70 days post SE). Tiagabine has been shown, in several experimental models, to be anticonvulsant and to prevent tonic-clonic seizures in the pilocarpine model [30], in the perforant pathway stimulation model, in audiogenic seizures [31], genetically prone epilepsy strains [32,33], in the maximum electric shock model, in the PTZ model [14], the electric kindling model [34,35], and in seizures induced by dendrotoxin [36]. However, it has been shown that the selective increase in this type of tonic-mediated inhibition is a sufficient condition to induce non-convulsive seizures in genetically and pharmacological absence epilepsy models [37].

Thus, data presented in this paper showed that Parawixin2 treatment in the lithium-pilocarpine model suggests this compound presents a mechanism that could be promising for TLE treatment in the chronic period.

### 3.2. Neuroprotective Effects of Parawixin2 and Tiagabine

Neuronal loss is an aspect that has gained much attention in the epilepsy research field. Nevertheless, it is well known that most drugs that inhibit neuronal loss do not have an antiepileptogenic effect [38].

In this work, we show that Parawixin2 administration results in changes in neuronal density in the CA3 and hilus layers, even when the administration is in the late phase of the chronic epilepsy model. This is the first report demonstrating the neuroprotective effects of Parawixin2 in a chronic epilepsy model. The acute neuroprotective effects of Parawixin2 have been shown experimentally in a glaucoma model [20]. Tiagabine treatment resulted in significant protection of the CA3 layer, by preventing neuronal density reduction. Similarly, Tiagabine neuroprotective effects in CA1 and CA3c layers have already been shown in the perforant *pathway* stimulation model [30]. Tiagabine is also neuroprotective in other neuronal injury models such as glucose/oxygen in vitro deprivation [39], in vivo global transient ischemic injury [40,41] and in the Huntigton transgenic mice N171-82Q model [42].

In this study we observed—in the hilus layer—that vehicle-treated animals had intense FJC+ staining even 70 days after SE, and that both Parawixin2 and Tiagabine decreased neurodegeneration, as seen by reduction in FJC+ staining. Other reports have demonstrated there is an intense FJC staining 60 days after SE induction, [25,43]. In the chronic phase of the pilocarpine model, a great percentage (approximately 80%) of FJC+ staining can still be observed and co-localized with GABAergic neurons stained with Somatostatin, Neuropeptide Y [44] and GAD-67 [44]. This implies that the inhibitory system might be severely compromised after SE. Also FJC+ stainning, evaluated in a long scale time course, can provide a better comprehension of the underlying pathological alterations in continuous epileptogenic process and spontaneous recurrent seizure [44].

### 3.3. Parvalbuminergic Cell Loss in the Chronic Phase of Pilocarpine Model and Effects of Parawixin2 Treatment

Groups subjected to SE and treated with vehicle or Tiagabine showed a reduction in the number of PV-ir in the granular layer of the DG. During the chronic period, PV interneuron loss is directedly linked to seizure frequency and severity [8]. In TLE patients, there is a decrease in number and intensity of PV immunostained fibers in granular DG—presumably inhibitory pericelullar fibers—suggesting that chandelier and basket cells might also have decreased [7]. Likewise, it was reported that PV-ir cells are reduced on the hilus and CA3 layers in the human epileptic hippocampus [45]. It has been proposed that PV focal loss in TLE patients is related to the onset and maintenance of epileptic activity [9].

The death of interneurons’ population has recently been widely studied, and it was found that there is also a great interneurons loss that is mostly GABAergic [4]. It was demonstrated that, in hippocampal slices from rats subjected to pilocarpine TLE model, epileptiform activity is independent of N-Methyl-D-Aspartate (NMDA) receptors, and inhibitory postsynaptic potential mediated by GABA_A_ receptors has reduced amplitude and frequency. These results suggest that hyperexcitability of pilocarpine-treated animals may result from the loss of interneurons in the deep layers of the hippocampus [43] and in the entorhinal cortex [46].

Therefore, strong evidence has supported PV-ir decrease related to epileptogenic activity in the hippocampus, and established a potential cell subpopulation, at which therapy should aim [8]. We observed a significant PV-ir reduction in epileptic animals, and that Parawixin2 had a significant effect in preserving those PV interneurons, particularly in the CA3 and DG layers. Complemetary to that, Parawixin2 group presented PV-ir cells’ preserved morphology, which appeared to be permeating the granular layer and the hilus of the DG, in comparison with vehicle-treated animals. Thus, this data supports potentially targeting these PV interneurons for neuroprotection and seizure control when estabilishing an antiepileptic treatment.

Interestingly, it was recently shown that closed-loop, on-demand stimulation of PV cells during SRS onset efficiently blocked seizures, and an acute intervention of closed-loop, on-demand stimulation reduced the frequency and severity of successive seizures. Collectively, these studies are important contributions to a further understanding of basic epilepsy mechanisms, by identifying cell populations that specifically orient pharmacological interventions [47].

Surprisingly, PV staining in Parawixin2 treatment was significantly different from vehicle and Tiagabine groups, and also from the naïve group (not subjected to SE). There is evidence that the epileptogenic process may induce an antigen up-regulation once there is interneuron axonal sprouting in epileptic patients [48,49], and in rodent models [50]. Therefore, Parawixin2 treatment could have enhanced a latent antigen up-regulation.

Recent reports showed that the initial GABAergic epileptogenic circuitry has a great impact on interneuron loss by reducing GABAergic synapses, however, in the chronic period, synaptogenesis may overcome basal levels. Because of this finding, it is believed that even though there are a great number of inhibitory synapses in epileptic circuitry, there is a functional reduction of GABA release in granular cells. Therefore—in the chronic phase—one might expect the opposite, with normal levels of GABAergic synapses but abundant non-functional presence, and that the reestablishment of normal function of these newly formed synapses in the epileptic tissue may imply anticonvulsant properties [51].

## 4. Conclusions

Parawixin2— shown in previous studies to be an inhibitor of GABA transport—exerts an antiepileptic effect in the chronic phase of the pilocarpine model. Additionally, it results in a neuroprotective effect as shown by decreased neurodegeneration in important cell populations such as Parvabuminergic interneurons. This work, along with previous work from our research group, reinforces the great and unexplored therapeutic potential of neuroactive compounds from arthropods venoms. This study highlights the importance of evaluating the subtypes of GABA transporters in epileptogenesis and to which subtype Parawixin2 interacts. To conclude, we propose that Parawixin2 has a potential as a framework molecule for drug development for disease-modifying agents for epilepsy treatment.

## 5. Methods

### 5.1. Animals

Male Wistar rats, weighing between 220 g and 250 g at the beginning of the experiment (*n* = 30), were purchased from the University of São Paulo Central Animal Facility in Ribeirão Preto Campus. These animals were housed in pairs at the Biology Department, and maintained under ventilation and controlled temperature (25 °C ± 2 °C) with dark/light circle of 12 h (light turned on at 7:00 a.m.), with free access to water and food. All protocols followed the ethical principles in animal experimentation. This study was approved by the University Ethics Committee for Animal Experimentation (CEUA, Campus USP/RP) under the protocol number 11.1.1389.53.2.

### 5.2. High Perfomance Liquid Cromatography and Dereplication

Parawixin2 was isolated from the venom of *Parawixia bistriata* spider using Liquid Chromatography, as previously described [15], with minor modifications. Briefly, *Parawixia bistrata* spider specimens were collected in the region of Ribeirão Preto (S.P, Brazil) and immediately frozen at −20 °C for further extraction of venom glands. Venom glands were extracted with the aid of tweezers and ophthalmic scissors, then homogenized in deionized water and centrifuged at 10,621× g for 3 min at 4 °C.

For the first fractionation, crude venom from ~6000 venom glands was solubilized in deionized water/acetonitrile (ACN) (9:1, *v*/*v*) and ultrafiltered using a 2000 Da Microcon^®^ filter (Millipore, Billerica, MA, USA). The filtered material was then lyophilized and weighed. The material (240 mg) was then solubilized in 20 mL of H_2_O/ACN 9:1 (v:v, filtered through a cellulose acetate membrane 0.45 μm, Millipore). 1 mL aliquots were injected into HPLC-DAD coupled to a Shimadzu C-18 reversed phase column (250 mm × 20 mm, 5 μm particles, Shimadzu Scientific Instruments, Sydney, NW, Australia) for preparative scale fractioning. In the second fractionation, the elution program used was the infusion of 10 min 0.05% TFA/H_2_O at 1.0 mL/min flow.

High-resolution electrospray ionization quadrupole time-of-flight (ESI-MS) spectrum and tandem mass spectrometry (ESI-MS/MS) were acquired on an UltrOTOF apparatus (Bruker Daltonics, Billerica, MA, USA). Solutions were infused into the ESI source using syringe pump (Harvard Apparatus, Holliston, MA, USA), at a flow rate of 10 L/min. Collision-induced dissociation was performed on the isolated protonated molecule using N_2_ as collision gas and 15 eV as collisional energy.

The compound was purified from P.B. fractions using essentially the same process reported by Beleboni et al. (2006). The identity of the compound was thus confirmed by comparison of retention times, UV, ESI-HRMS and ESI-MS/MS spectra, with the compound previously isolated. ESI-HRMS afforded [M + H] ^+^ at *m*/*z* 175.1196, giving us the molecular formula C_6_H_15_N_4_O_2_ with a 0.6 ppm error. ESI-MS/MS fragmentation of *m*/*z* 175 produced essentially the same fragmentation pattern reported by Beleboni et al. (2006), to be noted, the diagnostic product ions *m*/*z* 158 (loss of NH_3_), *m*/*z* 130 (loss of CONH_3_), *m*/*z* 116 (loss of NHCONH_2_) and *m*/*z* 60 (NHCONH_3_^+^), thus confirming the compound identity.

Purity was estimated by UV and MS/MS, which presented no other ions except those produced by Parawixin 2 by in source dissociation, and by ESI-MS, in which the isolated compound presented a single majoritary peak.

The compound of interest was examined for modulation of GABA transport activity in synaptosomes preparared from rat brain cortex, as previously described [17].

### 5.3. Drugs

Compounds lithium chloride (Sigma, St. Louis, MO, USA) and methylscopolamine (Tocris Bioscience, Bristol, UK) were administered subcutaneously (s.c). Pilocarpine hydrochloride (Sigma, St. Louis, MO, USA), sodium thiopental (Cristália, Minas Gerais, Brazil), and atropine (UCB, Brazil) were administred intraperitoneally (i.p).

Deionized water as vehicle (Vehicle), Tiagabine (TGB, Sigma, St. Louis, MO, USA) or Parawixin2 were administered intracerebroventriculary (i.c.v).

### 5.4. Status Epilepticus Induction

In our model, rats were injected with lithium chloride (127 mg/kg) 18–20 h prior to a subcutaneous (s.c) injection of pilocarpine hydrochloride (30 mg/kg). Also, in order to prevent peripheral cholinergic effects, animals received the cholinergic antagonist methylscopolamine (1 mg/kg, s.c) 30 min before pilocarpine injection. After 90 min of *status epilepticus* (SE) establishment, animals received thiopental sodium by i.p injection (30 mg/kg, i.p) to mitigate the seizures. The assessment of behavioral seizures was based on Racine limbic seizures classification [52] and SE criteria corresponded to the time when animal started to present sustained behaviors or higher limbic seizures defined by score 3, with no spontaneous recovery [53]. To avoid false-positive results, animals that had not reached SE or ceased it spontaneously were excluded from this study. Additionally, our study included two healthy control groups: one that did not undergo surgical procedure or SE (the Naïve group), and a group treated with lithium and methylscopolamine (Li + Me).

### 5.5. Spontaneous Recurrent Seizures Monitoring

To monitor spontaneous recurrent seizures, digital cameras (Surveillance Colour Sensor Day and Night, Hi-resolution) were installed at a distance of approximately 50 cm from the home cages. These cameras were connected to a computer with Lux Vision software (Lux Vision, Ribeirão Preto, SP, Brazil) to acquire videos. Settings were adjusted to record videos daily during the period from 7:00 a.m. to 7:00 p.m. It is known that the seizure threshold is reduced during sleep (during the light phase of the circadian cycle, as rodents are nocturnal), therefore seizures are more likely to occur in this period [29]. Behavioral seizures were monitored and scored according to Racine [52].

### 5.6. Stereotactic Surgery

After 55 days of SE induction, animals were submitted to stereotactic surgery to implant a guide cannula in the left lateral ventricle. Before the surgery, atropine (UCB, Brazil) was administered (0.1 mg/kg). Animals were anaesthetized with thiopental sodium (40 mg/kg, i.p) and subsequently placed in a stereotaxic apparatus (Stoelting-Standard, Chicago, IL, USA). A local injection of 2% lidocaine hydrochloride (SS White, Rio de Janeiro, Brazil) was applied and then the skull was exposed. The guide cannula was implanted into the right lateral ventricle (AP-0.9 mm, ML-1.6 mm, DV-3.4 mm). This cannula consists of a segment of hypodermic needle of 25 mm × 7 mm (22 G), of 10 mm length and 0.7 mm outer diameter.

After the acrylic polymerization, the cannula was sealed with stainless steel wire (to prevent obstruction). After surgery, animals were housed and left undisturbed for a recovery period of 5 days.

### 5.7. Intracerebroventricular Treatment

After 60 days of SE induction, animals were randomly divided into 3 treatment groups that each received daily injections (i.c.v) of either 1 μL deionized water (Vehicle, *n* = 6), Tiagabine (TGB, *n* = 5) at 6.4 mM—a dose that was previously shown to selectively inhibit GAT-1 [54]—or Parawixin2 (Pwx2, *n* = 6) at 0.86 mM—a dose that was shown to be anticonvulsant and neuroprotective. Treatments were perfomed for 10 consecutive days, at the same time (between 12:00 p.m. and 2:00 p.m.).

### 5.8. Tissue Preparation and Histology

One day after the last treatment, animals were euthanized by deep anesthesia with thiopental sodium (120 mg/kg, i.p), followed by a transcardiac perfusion with an influx of sodium phosphate buffer solution (0.05 M PBS, pH 7.4) followed by 4% paraformaldehyde solution (PFA diluted in 0.05 M PBS, pH 7.4; Synth, Brazil), through the cardiac left ventricle. Subsequently, brains were cryoprotected in 30% sucrose (diluted in 0.05 M PBS) for 24 h at 4 °C.

A cryostat (Leica Microsystems, Wetzlar, Germany) was used to section the frozen brains at 20 μm thick between −3.14 and −4.3 mm anteroposterior direction from bregma [55]. Hippocampal sections were mounted on three glass slides and maintained at −20 °C.

### 5.9. Nissl Staining

Nissl staining was performed to visualize cell nuclei, making it possible to estimate neuronal density.

Briefly, sections were first dehydrated. Slides were consecutively rinsed in 95, 80 and 70% ethanol solutions (*v*/*v*, in distilled water) and then rinsed in water. Slides were then rinsed in cresyl violet solution (0.5%) for 30 min and then washed with distilled water. Subsequently, sections were dehydrated by consecutive washes of increased concentrations of ethanol solution and xylene. Slides were covered with glass coverslips and Permount (Fisher Scientific, Hampton, NH, USA) as a mounting medium.

### 5.10. Detection of Neuronal Degeneration with Fluoro-Jade C

Fluoro-Jade C is a fluorochrome that stains neuronal degeneration and has been used in many models of neurodegeneration, including TLE models. Briefly, slides were incubated in 0.06% potassium permanganate solution, followed by incubation with 0.001% Fluoro-Jade C solution (Millipore, Billerica, MA, USA) for 30 min, followed by washes and covering with glass coverslips, using Fluoromount (Millipore, Billerica, MA, USA) as a fluorescent mounting medium [56].

### 5.11. Parvalbumin Immunostaining

Briefly, slides were washed in PBS (10 mM), then glycine (0.1 M, diluted in PBS 10 M) and then in PBS-T (0.1% Triton X-100; Sigma, St. Louis, MO, USA). Sections were incubated with bovine serum albumin Fraction V (Sigma, St. Louis, MO, USA) in PBS-T and then with anti-parvalbumin primary antibody (1:2000; Millipore, Billerica, MA, USA) overnight. Afterwards, slides were washed in PBS, and incubated with secondary antibody conjugated to fluorophore (Alexa-594; 1:1000; Invitrogen, Carlsbad, CA, USA) for 2 h. Slides were then washed in PBS and mounted with Fluoroshield medium with DAPI (Sigma, St. Louis, MO, USA).

### 5.12. Quantitative Analysis of Neuronal Damage

For each section, a total of ten images were captured from the entire length of the pyramidal layers of CA1, CA3, DG and hilus. Images were captured with a digital camera (DFC300 FX, Leica Microsystems, Wetzlar, Germany) connected to a microscope (DM 5000 B, Leica Microsystems, Wetzlar, Germany), and to a computer. The images for cell counting and area measurement were captured using 400× magnification for CA1, CA3 and DG layers, and 200× for the hilus. Viable neurons, nuclei diameters and areas were quantified manually with Q-Win software (Leica Microsystems, Wetzlar, Germany).

The density of uninjured cells (n) was determined by averaging the values obtained from the hippocampus sections analyses for each individual in each group. Cell density was calculated using Abercrombie correction method: *N* (mm^2^) = *n* [T/(T + D)]/A; where *N* is the cell density, T is section thickness (20 μm), D is the nucleus diameter and A represents the measurement (μm^2^) for each area of hippocampal region [57].

Immunofluorescence for Flouro-Jade C was analyzed by counting all immunoreactive cells (counted regardless staining intensity) for each area of the hippocampal region.

Parvalbumin staining was analyzed by counting all immunoreactive cells in six regions of interest (ROI) with an area of 0.079 mm^2^ in CA1, CA3 and DG regions; and 0.288 mm^2^ in hilus region.

### 5.13. Qualitative Analysis of Neuronal Damage

Qualitative histopathological analysis was performed by evaluating morphological parameters—such as cell integrity in hippocampal layers—to determine morphological changes compatible with histopathological features. Vacuolalization was determined when cells showed weak Nissl corpuscles staining in the central portion of the cell and displacement of the nucleus to a periphery. Gliosis is characterized by high density of nucleus of glia. Hippocampal layers are very well defined and layer disorganization was also analysed. Finally, injured neurons usually shrink, become eosinophilic due to condensation of mitochondria, and their nuclei become pyknotic, so these parameters were also analyzed.

### 5.14. Statistical Analysis

To characterize mean frequency, duration and score of recurrent seizures during the whole period, data were analyzed using the one-way ANOVA test followed by the post hoc Student Newman Keuls test. To analyze daily seizure frequency, duration, and score, two-way ANOVA, followed by Bonferroni test (with time and treatment as factors) was used. Data obtained from histological measurements were analyzed using one-way ANOVA followed by post hoc Student-Newman Keuls test, with *p* < 0.05 considered statistically significant. All analyses were performed with Prism6 software from Graph Pad, version 11.5 (La Jolla, San Diego, CA, USA).

## Supplementary Materials

The following are available online at www.mdpi.com/2072-6651/9/9/262/s1, Table S1: Daily seizure frequency post hoc analysis for treatment and time factors, respectively.
