# Supplementary material for: Disease Modifying Effects of the Spider Toxin Parawixin2 in the Experimental Epilepsy Model
Source: Toxins (Basel). 2017 Aug 25;9(9):262. doi: 10.3390/toxins9090262 (PMC5618195; doi:10.3390/toxins9090262)
Supplement: Supplementary file 1 [file toxins-09-00262-s001.pdf]

Supplementary Materials: Disease Modifying Effects of the Spider Toxin Parawixin2 in the Experimental Epilepsy Model

Lívea Dornela Godoy, Jose Luiz Liberato, Marcus V. B. Celani, Leonardo Gobbo-Neto, Norberto Peporine Lopes and Wagner Ferreira dos Santos

Table S1. Daily seizure frequency post hoc analysis for treatment and time factors, respectively.

Tukey's Multiple Comparisons Test	Mean Diff.	95% CI of Diff.	Significant?	Summary	Adjusted *P* Value
day 1					
SE+TGB vs. SE+Vehicle	0.6	−1.828 to 3.028	No	ns	0.8276
SE+Pwx2 vs. SE+Vehicle	−2.6	−5.028 to −0.1721	Yes	*	0.0328
SE+Pwx2 vs. SE+TGB	−3.2	−5.628 to −0.7721	Yes	**	0.0062
day 2					
SE+TGB vs. SE+Vehicle	−1.8	−4.228 to 0.6279	No	ns	0.1878
SE+Pwx2 vs. SE+Vehicle	−2	−4.428 to 0.4279	No	ns	0.128
SE+Pwx2 vs. SE+TGB	−0.2	−2.628 to 2.228	No	ns	0.9792
day 3					
SE+TGB vs. SE+Vehicle	−1.2	−3.628 to 1.228	No	ns	0.4716
SE+Pwx2 vs. SE+Vehicle	−2.6	−5.028 to −0.1721	Yes	*	0.0328
SE+Pwx2 vs. SE+TGB	−1.4	−3.828 to 1.028	No	ns	0.3607
day 4					
	−1.4	−3.828 to 1.028	No	ns	0.3607
SE+Pwx2 vs. SE+Vehicle	−1.6	−4.028 to 0.8279	No	ns	0.2653
SE+Pwx2 vs. SE+TGB	−0.2	−2.628 to 2.228	No	ns	0.9792
day 5					
SE+TGB vs. SE+Vehicle	0.4	−2.028 to 2.828	No	ns	0.9193
SE+Pwx2 vs. SE+Vehicle	−1.2	−3.628 to 1.228	No	ns	0.4716
SE+Pwx2 vs. SE+TGB	−1.6	−4.028 to 0.8279	No	ns	0.2653
day 6					
SE+TGB vs. SE+Vehicle	−0.2	−2.628 to 2.228	No	ns	0.9792
SE+Pwx2 vs. SE+Vehicle	−1	−3.428 to 1.428	No	ns	0.5925
SE+Pwx2 vs. SE+TGB	−0.8	−3.228 to 1.628	No	ns	0.7148
day 7					
SE+TGB vs. SE+Vehicle	0.4	−2.028 to 2.828	No	ns	0.9193
SE+Pwx2 vs. SE+Vehicle	−0.2	−2.628 to 2.228	No	ns	0.9792
SE+Pwx2 vs. SE+TGB	−0.6	−3.028 to 1.828	No	ns	0.8276
day 8					
SE+TGB vs. SE+Vehicle	−1.8	−4.228 to 0.6279	No	ns	0.1878
SE+Pwx2 vs. SE+Vehicle	−2.8	−5.228 to −0.3721	Yes	*	0.0194
SE+Pwx2 vs. SE+TGB	−1	−3.428 to 1.428	No	ns	0.5925
day 9					
SE+TGB vs. SE+Vehicle	0.4	−2.028 to 2.828	No	ns	0.9193
SE+Pwx2 vs. SE+Vehicle	0.6	−1.828 to 3.028	No	ns	0.8276
SE+Pwx2 vs. SE+TGB	0.2	−2.228 to 2.628	No	ns	0.9792
day 10					
SE+TGB vs. SE+Vehicle	−1.4	−3.828 to 1.028	No	ns	0.3607
SE+Pwx2 vs. SE+Vehicle	−1.4	−3.828 to 1.028	No	ns	0.3607
SE+Pwx2 vs. SE+TGB	0	−2.428 to 2.428	No	ns	>0.9999
SE+Vehicle					
day 2 vs. 1	0.2	−2.681 to 3.081	No	ns	>0.9999
day 3 vs. 1	−0.4	−3.281 to 2.481	No	ns	>0.9999
day 4 vs. 1	−1.6	−4.481 to 1.281	No	ns	0.7374
day 5 vs. 1	−3	−5.881 to −0.1188	Yes	*	0.0342
day 6 vs. 1	−2.8	−5.681 to 0.08119	No	ns	0.0642
day 7 vs. 1	−3	−5.881 to −0.1188	Yes	*	0.0342
day 8 vs. 1	0.2	−2.681 to 3.081	No	ns	>0.9999
day 9 vs. 1	−3	−5.881 to −0.1188	Yes	*	0.0342
day 10 vs. 1	−1.6	−4.481 to 1.281	No	ns	0.7374
day 3 vs. 2	−0.6	−3.481 to 2.281	No	ns	0.9996
day 4 vs. 2	−1.8	−4.681 to 1.081	No	ns	0.5882

Table S1. *Cont.*

Tukey's Multiple Comparisons Test	Mean Diff.	95% CI of Diff.	Significant?	Summary	Adjusted *P* Value
day 5 vs. 2	−3.2	−6.081 to −0.3188	Yes	*	0.0173
day 6 vs. 2	−3	−5.881 to −0.1188	Yes	*	0.0342
day 7 vs. 2	−3.2	−6.081 to −0.3188	Yes	*	0.0173
day 8 vs. 2	0	−2.881 to 2.881	No	ns	>0.9999
day 9 vs. 2	−3.2	−6.081 to −0.3188	Yes	*	0.0173
day 10 vs. 2	−1.8	−4.681 to 1.081	No	ns	0.5882
day 4 vs. 3	−1.2	−4.081 to 1.681	No	ns	0.9404
day 5 vs. 3	−2.6	−5.481 to 0.2812	No	ns	0.114
day 6 vs. 3	−2.4	−5.281 to 0.4812	No	ns	0.1905
day 7 vs. 3	−2.6	−5.481 to 0.2812	No	ns	0.114
day 8 vs. 3	0.6	−2.281 to 3.481	No	ns	0.9996
day 9 vs. 3	−2.6	−5.481 to 0.2812	No	ns	0.114
day 10 vs. 3	−1.2	−4.081 to 1.681	No	ns	0.9404
day 5 vs. 4	−1.4	−4.281 to 1.481	No	ns	0.8595
day 6 vs. 4	−1.2	−4.081 to 1.681	No	ns	0.9404
day 7 vs. 4	−1.4	−4.281 to 1.481	No	ns	0.8595
day 8 vs. 4	1.8	−1.081 to 4.681	No	ns	0.5882
day 9 vs. 4	−1.4	−4.281 to 1.481	No	ns	0.8595
day 10 vs. 4	0	−2.881 to 2.881	No	ns	>0.9999
day 6 vs. 5	0.2	−2.681 to 3.081	No	ns	>0.9999
day 7 vs. 5	0	−2.881 to 2.881	No	ns	>0.9999
day 8 vs. 5	3.2	0.3188 to 6.081	Yes	*	0.0173
day 9 vs. 5	0	−2.881 to 2.881	No	ns	>0.9999
day 10 vs. 5	1.4	−1.481 to 4.281	No	ns	0.8595
day 7 vs. 6	−0.2	−3.081 to 2.681	No	ns	>0.9999
day 8 vs. 6	3	0.1188 to 5.881	Yes	*	0.0342
day 9 vs. 6	−0.2	−3.081 to 2.681	No	ns	>0.9999
day 10 vs. 6	1.2	−1.681 to 4.081	No	ns	0.9404
day 8 vs. 7	3.2	0.3188 to 6.081	Yes	*	0.0173
day 9 vs. 7	0	−2.881 to 2.881	No	ns	>0.9999
day 10 vs. 7	1.4	−1.481 to 4.281	No	ns	0.8595
day 9 vs. 8	−3.2	−6.081 to −0.3188	Yes	*	0.0173
day 10 vs. 8	−1.8	−4.681 to 1.081	No	ns	0.5882
day 10 vs. 9	1.4	−1.481 to 4.281	No	ns	0.8595
SE+TGB					
day 2 vs. 1	−2.2	−5.081 to 0.6812	No	ns	0.2982
day 3 vs. 1	−2.2	−5.081 to 0.6812	No	ns	0.2982
day 4 vs. 1	−3.6	−6.481 to −0.7188	Yes	**	0.0039
day 5 vs. 1	−3.2	−6.081 to −0.3188	Yes	*	0.0173
day 6 vs. 1	−3.6	−6.481 to −0.7188	Yes	**	0.0039
day 7 vs. 1	−3.2	−6.081 to −0.3188	Yes	*	0.0173
day 8 vs. 1	−2.2	−5.081 to 0.6812	No	ns	0.2982
day 9 vs. 1	−3.2	−6.081 to −0.3188	Yes	*	0.0173
day 10 vs. 1	−3.6	−6.481 to −0.7188	Yes	**	0.0039
day 3 vs. 2	0	−2.881 to 2.881	No	ns	>0.9999
day 4 vs. 2	−1.4	−4.281 to 1.481	No	ns	0.8595
day 5 vs. 2	−1	−3.881 to 1.881	No	ns	0.9815
day 6 vs. 2	−1.4	−4.281 to 1.481	No	ns	0.8595
day 7 vs. 2	−1	−3.881 to 1.881	No	ns	0.9815
day 8 vs. 2	0	−2.881 to 2.881	No	ns	>0.9999
day 9 vs. 2	−1	−3.881 to 1.881	No	ns	0.9815
day 10 vs. 2	−1.4	−4.281 to 1.481	No	ns	0.8595
day 4 vs. 3	−1.4	−4.281 to 1.481	No	ns	0.8595
day 5 vs. 3	−1	−3.881 to 1.881	No	ns	0.9815
day 6 vs. 3	−1.4	−4.281 to 1.481	No	ns	0.8595
day 7 vs. 3	−1	−3.881 to 1.881	No	ns	0.9815
day 8 vs. 3	0	−2.881 to 2.881	No	ns	>0.9999
day 9 vs. 3	−1	−3.881 to 1.881	No	ns	0.9815
day 10 vs. 3	−1.4	−4.281 to 1.481	No	ns	0.8595
day 5 vs. 4	0.4	−2.481 to 3.281	No	ns	>0.9999
day 6 vs. 4	0	−2.881 to 2.881	No	ns	>0.9999
day 7 vs. 4	0.4	−2.481 to 3.281	No	ns	>0.9999

Table S1. *Cont.*

Tukey's Multiple Comparisons Test	Mean Diff.	95% CI of Diff.	Significant?	Summary	Adjusted *P* Value
day 8 vs. 4	1.4	−1.481 to 4.281	No	ns	0.8595
day 9 vs. 4	0.4	−2.481 to 3.281	No	ns	>0.9999
day 10 vs. 4	0	−2.881 to 2.881	No	ns	>0.9999
day 6 vs. 5	−0.4	−3.281 to 2.481	No	ns	>0.9999
day 7 vs. 5	0	−2.881 to 2.881	No	ns	>0.9999
day 8 vs. 5	1	−1.881 to 3.881	No	ns	0.9815
day 9 vs. 5	0	−2.881 to 2.881	No	ns	>0.9999
day 10 vs. 5	−0.4	−3.281 to 2.481	No	ns	>0.9999
day 7 vs. 6	0.4	−2.481 to 3.281	No	ns	>0.9999
day 8 vs. 6	1.4	−1.481 to 4.281	No	ns	0.8595
day 9 vs. 6	0.4	−2.481 to 3.281	No	ns	>0.9999
day 10 vs. 6	0	−2.881 to 2.881	No	ns	>0.9999
day 8 vs. 7	1	−1.881 to 3.881	No	ns	0.9815
day 9 vs. 7	0	−2.881 to 2.881	No	ns	>0.9999
day 10 vs. 7	−0.4	−3.281 to 2.481	No	ns	>0.9999
day 9 vs. 8	−1	−3.881 to 1.881	No	ns	0.9815
day 10 vs. 8	−1.4	−4.281 to 1.481	No	ns	0.8595
day 10 vs. 9	−0.4	−3.281 to 2.481	No	ns	>0.9999
SE+Pwx2					
day 2 vs. 1	0.8	−2.081 to 3.681	No	ns	0.9963
day 3 vs. 1	−0.4	−3.281 to 2.481	No	ns	>0.9999
day 4 vs. 1	−0.6	−3.481 to 2.281	No	ns	0.9996
day 5 vs. 1	−1.6	−4.481 to 1.281	No	ns	0.7374
day 6 vs. 1	−1.2	−4.081 to 1.681	No	ns	0.9404
day 7 vs. 1	−0.6	−3.481 to 2.281	No	ns	0.9996
day 8 vs. 1	0	−2.881 to 2.881	No	ns	>0.9999
day 9 vs. 1	0.2	−2.681 to 3.081	No	ns	>0.9999
day 10 vs. 1	−0.4	−3.281 to 2.481	No	ns	>0.9999
day 3 vs. 2	−1.2	−4.081 to 1.681	No	ns	0.9404
day 4 vs. 2	−1.4	−4.281 to 1.481	No	ns	0.8595
day 5 vs. 2	−2.4	−5.281 to 0.4812	No	ns	0.1905
day 6 vs. 2	−2	−4.881 to 0.8812	No	ns	0.4348
day 7 vs. 2	−1.4	−4.281 to 1.481	No	ns	0.8595
day 8 vs. 2	−0.8	−3.681 to 2.081	No	ns	0.9963
day 9 vs. 2	−0.6	−3.481 to 2.281	No	ns	0.9996
day 10 vs. 2	−1.2	−4.081 to 1.681	No	ns	0.9404
day 4 vs. 3	−0.2	−3.081 to 2.681	No	ns	>0.9999
day 5 vs. 3	−1.2	−4.081 to 1.681	No	ns	0.9404
day 6 vs. 3	−0.8	−3.681 to 2.081	No	ns	0.9963
day 7 vs. 3	−0.2	−3.081 to 2.681	No	ns	>0.9999
day 8 vs. 3	0.4	−2.481 to 3.281	No	ns	>0.9999
day 9 vs. 3	0.6	−2.281 to 3.481	No	ns	0.9996
day 10 vs. 3	0	−2.881 to 2.881	No	ns	>0.9999
day 5 vs. 4	−1	−3.881 to 1.881	No	ns	0.9815
day 6 vs. 4	−0.6	−3.481 to 2.281	No	ns	0.9996
day 7 vs. 4	0	−2.881 to 2.881	No	ns	>0.9999
day 8 vs. 4	0.6	−2.281 to 3.481	No	ns	0.9996
day 9 vs. 4	0.8	−2.081 to 3.681	No	ns	0.9963
day 10 vs. 4	0.2	−2.681 to 3.081	No	ns	>0.9999
day 6 vs. 5	0.4	−2.481 to 3.281	No	ns	>0.9999
day 7 vs. 5	1	−1.881 to 3.881	No	ns	0.9815
day 8 vs. 5	1.6	−1.281 to 4.481	No	ns	0.7374
day 9 vs. 5	1.8	−1.081 to 4.681	No	ns	0.5882
day 10 vs. 5	1.2	−1.681 to 4.081	No	ns	0.9404
day 7 vs. 6	0.6	−2.281 to 3.481	No	ns	0.9996
day 8 vs. 6	1.2	−1.681 to 4.081	No	ns	0.9404
day 9 vs. 6	1.4	−1.481 to 4.281	No	ns	0.8595
day 10 vs. 6	0.8	−2.081 to 3.681	No	ns	0.9963
day 8 vs. 7	0.6	−2.281 to 3.481	No	ns	0.9996
day 9 vs. 7	0.8	−2.081 to 3.681	No	ns	0.9963
day 10 vs. 7	0.2	−2.681 to 3.081	No	ns	>0.9999
day 9 vs. 8	0.2	−2.681 to 3.081	No	ns	>0.9999
day 10 vs. 8	−0.4	−3.281 to 2.481	No	ns	>0.9999
day 10 vs. 9	−0.6	−3.481 to 2.281	No	ns	0.9996
